# NTPDase1 Modulates Smooth Muscle Contraction in Mice Bladder by Regulating Nucleotide Receptor Activation Distinctly in Male and Female

**DOI:** 10.3390/biom11020147

**Published:** 2021-01-23

**Authors:** Romuald Brice Babou Kammoe, Gilles Kauffenstein, Julie Pelletier, Bernard Robaye, Jean Sévigny

**Affiliations:** 1 Centre de Recherche du CHU de Québec, Université Laval, Québec City, QC G1V 4G2, Canada; Romuald.Babou@crchudequebec.ulaval.ca (R.B.B.K.); kauffenstein@unistra.fr (G.K.); Julie.Pelletier@crchudequebec.ulaval.ca (J.P.); 2 Département de Microbiologie-Infectiologie et d’immunologie, Faculté de Médecine, Université Laval, Québec City, QC G1V 0A6, Canada; 3 UMR INSERM 1260, Centre de Recherche en Biomédecine de Strasbourg, Université de Strasbourg, 67084 Strasbourg, France; 4 Institut de Recherche Interdisciplinaire en Biologie Humaine et Moléculaire, Université Libre de Bruxelles, 10 rue Adrienne Bolland, 6041 Gosselies, Belgium; Bernard.Robaye@ulb.be

**Keywords:** NTPDase1, extracellular nucleotides, bladder, smooth muscle cells, contraction, P2Y_6_ receptor, sex

## Abstract

Nucleotides released by smooth muscle cells (SMCs) and by innervating nerve terminals activate specific P2 receptors and modulate bladder contraction. We hypothesized that cell surface enzymes regulate SMC contraction in mice bladder by controlling the concentration of nucleotides. We showed by immunohistochemistry, enzymatic histochemistry, and biochemical activities that nucleoside triphosphate diphosphohydrolase-1 (NTPDase1) and ecto-5′-nucleotidase were the major ectonucleotidases expressed by SMCs in the bladder. RT-qPCR revealed that, among the nucleotide receptors, there was higher expression of P2X1, P2Y_1_, and P2Y_6_ receptors. Ex vivo, nucleotides induced a more potent contraction of bladder strips isolated from NTPDase1 deficient (*Entpd1*^−/−^) mice compared to wild type controls. The strongest responses were obtained with uridine 5′-triphosphate (UTP) and uridine 5′-diphosphate (UDP), suggesting the involvement of P2Y_6_ receptors, which was confirmed with *P2ry6***^−/−^** bladder strips. Interestingly, this response was reduced in female bladders. Our results also suggest the participation of P2X1, P2Y_2_ and/or P2Y_4_, and P2Y_12_ in these contractions. A reduced response to the thromboxane analogue U46619 was also observed in wild type, *Entpd1***^−/−^**, and *P2ry6***^−/−^** female bladders showing another difference due to sex. In summary, NTPDase1 modulates the activation of nucleotide receptors in mouse bladder SMCs, and contractions induced by P2Y_6_ receptor activation were weaker in female bladders.

## 1. Introduction

In addition to their role in cell metabolism, nucleotides and nucleosides are extracellular mediators that activate biological responses such as smooth muscle cell contraction [1]. Cells subjected to activation or mechanical stress release nucleotides in large quantities [2,3]. Virtually all cells are capable of releasing nucleotides in a controlled manner [4,5]. The mechanisms of nucleotide release have been the subject of intense research activities which revealed distinct pathways of release. For example, while activated platelets and neurons release nucleotides by exocytosis [6], nucleotide efflux occurs in neutrophils and T cells via pannexin-1 hemi-channels [7,8]. Certain cells also release nucleotides in a constitutive manner [9,10]. Smooth muscle cells (SMCs) from the walls of blood vessels can release up to 60% of their nucleotide content without affecting their viability [11]. This release occurs in response to stimulation by norepinephrine or by adenosine 5′-triphosphate (ATP) itself as when released by sympathetic nerve endings [11]. Prolonged exercise and hypoxia also trigger the release of nucleotides by cardiac and skeletal myocytes [12,13].

Once released, nucleotides activate P2X ionotropic receptors (P2X1-7) and P2Y metabotropic receptors (P2Y_1,2,4,6,11–14_) [4] coupled to G proteins, which are expressed in a ubiquitous manner [14,15]. The P2 receptor subtypes differ from one another according to their selectivity towards nucleotides [16,17,18]. While all P2X receptors are activated by ATP, P2Y_1_, P2Y_12_, and P2Y_13_ are activated by adenosine 5′-diphosphate (ADP), P2Y_2_ by ATP and uridine 5′-triphosphate (UTP), P2Y_4_ by UTP, P2Y_6_ by uridine 5′-diphosphate (UDP) (also by UTP in the mouse [19]), P2Y_11_ by ATP > ADP, and P2Y_14_ by UDP-glucose [20]. In addition, adenosine, which results from the complete dephosphorization of ATP and ADP by ectonucleotidases, activates P1 receptors, which are divided into four subtypes: A_1_, A_2A_, A_2B_, and A_3_ [21]. These receptors are widely distributed and their activation modulates a large number of biological processes, including the contraction of vascular smooth muscles [22]. ATP released as a co-transmitter with norepinephrine from sympathetic nerve endings causes calcium mobilization in vascular SMCs and vasoconstriction [23]. In contrast, activation of the adenosine A_2A_ receptor in these cells causes coronary vasodilation [24].

Nucleotide-induced responses can be modulated by cell surface enzymes, including members of the ecto-nucleoside triphosphate diphosphohydrolase (E-NTPDases) family [25]. These enzymes convert nucleosides tri- (ATP and UTP) and diphosphate (ADP and UDP) to their monophosphate derivative (adenosine 5′-monophosphate (AMP) and uridine 5′-monophosphate (UMP)) [26]. Hydrolysis thus terminates the nucleotide signal but can also allow the transient activation of P2 receptors activated by ADP or UDP [26]. Then, AMP is further hydrolyzed by ecto-5′-nucleotidase to generate adenosine, the ligand of P1 receptors [25,27]. Thus, these enzymes have the ability to control the relative concentrations of each nucleotide and nucleoside and, consequently, regulate the activation of P2 and P1 receptors and their effects [9]. The E-NTPDase family consists of eight members (NTPDase1-8), which have one or two transmembrane domains [28]. While NTPDase1, -2, -3, and -8 are linked to the plasma membrane with their active site facing the extracellular compartment, NTPDase4, -5, -6, and -7 are mainly located in the membrane of intracellular organelles [25]. Thus NTPDase1, -2, -3, and -8 can, in theory, regulate the activation of P2 receptors [26,29,30]. Importantly, each ectonucleotidase has distinct biochemical properties and affinity for the substrate, thus allowing fine regulation of the levels and effects of the nucleotides on their receptors [26]. Indeed, NTPDase1 effectively hydrolyzes ATP and ADP by producing AMP, NTPDase2 primarily hydrolyzes ATP and produces an accumulation of ADP while NTPDase3 and -8 act in an intermediate way between the two first cited [26].

Growing evidence suggests that extracellular nucleotides and NTPDase1 are involved in modulating the contraction of SMCs. We demonstrated that NTPDase1 is the ectonucleotidase responsible for the major part of the hydrolysis of nucleotides at the surface of vascular SMCs [19], its absence leading to a powerful contraction to UTP and UDP nucleotides via the activation of the P2Y_6_ receptor [19]. We also observed that NTPDase1 is expressed in SMCs of the intestine and vas deferens [31,32]. We reported that NTPDase1 was essential for maintaining expression and function of the P2X1 receptor in murine vas deferens and that its absence in *Entpd1*^−/−^ mice resulted in P2X1 desensitization, which hampered the contraction of the vas deferens resulting in reduced ejaculation and fertility [32].

The goal of the present work was to study the role of the dominant ectonucleotidase in mice bladder and to test whether this enzyme can modulate the contraction of this tissue. We observed that NTPDase1 was the major enzyme responsible for the hydrolysis of nucleotides at the surface of SMCs and that its absence resulted in an increased response to nucleotides which was different between male and female.

## 2. Materials and Methods

### 2.1. Materials

Paraformaldehyde (PFA), PMSF (phenylmethylsulfonyl fluoride), Tris-base, maleic acid, lead nitrate (Pb (NO_3_)_2_), manganese chloride (MnCl_2_), potassium chloride (KCl), ammonium sulfite ((NH_4_)_2_SO_3_, magnesium sulfate (MgSO_4_), 5-hydroxytryptamine (5HT), potato apyrase grade 7, adenosine 5′-triphosphate (ATP), adenosine 5′-diphosphate (ADP), uridine 5′-triphosphate (UTP), uridine 5′-diphosphate (UDP), and adenosine 5′-monophospahe (AMP) were purchased from Sigma–Aldrich (Oakville, ON, Canada). Thromboxane A2 analog (U46619) was purchased from Cayman Chemical (Ann Arbor, MI, USA). Sucrose and sodium chloride (NaCl) were obtained from Wisent (St Bruno, Canada). Calcium chloride (CaCl_2_) and dextrose were purchased from Fisher Scientific (Waltham, MT, USA). KH_2_PO_4_ and sodium bicarbonate (NaHCO_3_) were obtained from EMD Millipore (Mississauga, ON, Canada). Carbachol was purchased from Tocris Biosciences (Minneapolis, MN, USA). Cytokeratin 7 (Krt7) and smooth muscle myosin heavy chain 11 (Myh11) were purchased from Qiagen (Toronto, ON, Canada). Trizol was obtained from Invitrogen (Carlsbad, CA, United States). N’,N’-dimethyl-N-[4-[(E)-(3-methyl-1,3-benzothiazol-2-ylidene)methyl]-1-phenylquinolin-1-ium-2-yl]-N-propylpropane-1,3-diamine (SYBR Green) and DNAseI were from Roche Diagnostics (Indianapolis, IN, United States). Oligo (dt)_18_ was obtained from Fisher Scientific (Ottawa, ON, Canada). Antibodies against mouse NTPDase1 (mN1-2_c_) [33], mouse NTPDase2 (mN2-36_l_) [34], mouse NTPDase3 (mN3-3_c_) [33], mouse NTPDase8 (mN8-3_c_) [35], and rat ecto-5′-nucleotidase (rNu-9_l_) [36] were from http://ectonucleotidases-ab.com/.

### 2.2. Animals

All experiments were conducted in accordance with the guidelines of the Canadian Council on Animal Care, and protocols were approved by the Animal Care Committees of Université Laval. The protocol number 14-181 originally approved in February 2015 was renewed yearly and was updated to 19-010 in February 2019. Adult male and female C57BL/6 mice 12 to 16 weeks old (Charles River, Pointe-Claire, QC, Canada) were used as controls (65 wild type (WT) males and 44 WT females). NTPDase1-deficient (*Entpd1*^−/−^) mice were provided by Dr. S. C. Robson (BIDMC, HMS, Boston, MA) [30]. *Entpd1*^−/−^ mice, originally from the 129 SVJ×C57BL/6 background, were backcrossed 14 generations onto the C57BL/6 background. P2Y_6_-deficient (*P2ry6*^−/−^) mice obtained from B. Robaye (Université Libre de Bruxelles, Belgium) [37] were backcrossed 12 times with C57BL/6 mice from Charles River. A few backcrosses with wild type (WT) females were performed to ascertain that the mitochondrial DNA was the same in all mutant and control mice. Heterozygotes were then mated to obtained homozygote mice to generate a colony of both *P2ry6*^−/−^ and *Entpd1*^−/−^ mice. In our experiments, we used 46 *Entpd1*^−/−^ males, 30 *Entpd1*^−/−^ females, 15 *P2ry6*^−/−^ males, and 10 *P2ry6*^−/−^ females. Animals were maintained in a specific pathogen-free environment in a temperature-controlled room (21 °C) on a 12-h/12-h light and dark cycle and given unrestricted access to standard diet and tap water (or specified drinking solution).

### 2.3. Enzyme Histochemistry

Freshly dissected bladders from 16-week-old mice (3 WT males, 3 WT females, 3 *Entpd1*^−/−^ males, and 3 *Entpd1*^−/−^ females), sacrificed following isoflurane anesthesia, were embedded in optimal cutting temperature (OCT) freezing medium (Tissue-Tek, Sakura Finetek, Torrance, CA, USA), snap-frozen in isopentane in dry ice, and stored at −80 °C. Bladder sections (6 µm) obtained using a 2800 Frigocut-E Cryostat (Reicher-Jung, Leica Instruments GmbH, Deutschland, Germany) were prepared and routinely fixed in 10% phosphate-buffered formalin mixed with cold acetone (Fisher Scientific, Ottawa, ON, Canada) before further processing. Ectonucleotidase activities in bladder sections were localized using the Wachstein–Meisel lead phosphate precipitation method, as described [38]. Briefly, fixed tissue sections were preincubated for 30 min at 25 °C in Tris-maleate buffer (2 mM CaCl_2_, 250 mM sucrose, 50 mM Tris-maleate, pH 7.4). Enzymatic reaction for the hydrolysis of 100 µM nucleotides was performed for 40 min at 37 °C in the same buffer supplemented with 5 mM MnCl_2_, 2 mM Pb(NO_3_)_2_, and 3% dextran T250 (wt/vol). For control experiments, substrate was omitted. Reaction products were revealed by incubation of tissue sections with 1% (NH_4_)_2_SO_3_ (vol/vol) for 1 min. Sections were counterstained with aqueous hematoxylin, mounted in Mowiol mounting medium, and photographed under a microscope (model BX51, Olympus).

### 2.4. Immunohistochemistry

Sixteen-week-old mice (3 WT males, 3 WT females, 3 *Entpd1*^−/−^ males, and 3 *Entpd1*^−/−^ females) were sacrificed following isoflurane anesthesia. Bladder samples were excised, fixed in 4% paraformaldehyde for 4 h, immersed in sucrose overnight, and included in OCT freezing medium, sectioned, and processed for staining as previously described [33]. Briefly, 6 µm tissue sections were incubated overnight at 4 °C with the primary antibodies guinea pig anti-mouse NTPDase1 (mN1-2_c_) [33], rabbit anti-mouse NTPDase2 (mN2-36_l_) [34], guinea pig anti-mouse NTPDase3 (mN3-3_c_) [33], guinea pig anti-mouse NTPDase8 (mN8-3_c_) [35] or rabbit anti-rat ecto-5′-nucleotidase (rNu-9_l_) [36], and then with biotinylated goat anti-guinea pig or anti-rabbit secondary antibodies, accordingly, at 25 °C. Pre-immune sera were routinely included as controls. Sections were counterstained with aqueous hematoxylin, mounted in Mowiol mounting medium, and photographed under a microscope (model BX51, Olympus).

### 2.5. Nucleotidase Activity Measurement

Tissues from 16-week-old mice (11 WT males, 7 *Entpd1*^−/−^ males and 4 WT females), were homogenized with a polytron in the following buffer: 95 mM NaCl, 0.1 mM PMSF, and 45 mM Tris, pH 7.6. The homogenates were centrifuged at 3000 rpm for 5 min using a Mikro 200R centrifuge (Hettich Company, MT, USA) and the resulting supernatants were used for activity measurements. The enzyme activity of protein extract was measured at 37 °C in 1 mL of the reaction buffer constituted of 5 mM CaCl_2_, 80 mM Tris buffer pH 7.4 as described previously [39,40]. Briefly, 10 µl of protein at the appropriate dilution was added to the reaction buffer and was incubated for 3 min at 37 °C. The reaction was started by the addition of 0.5 mM substrate (ATP, ADP, UTP, UDP, or AMP). The reaction was stopped after 15 min by the addition of 250 µl of malachite green reagent, and the liberated inorganic phosphate (Pi) was measured at 630 nm according to the methodology described by Baykov et al. (1988) [40]. The results were expressed as nmoles Pi/min/mg protein. All experiments were performed in triplicate. For control or blank experiments, the diluted protein (10 µl) was added after stopping the reaction with malachite green.

### 2.6. Quantitative Real-Time PCR (RT-qPCR)

RT-qPCR was performed on bladder smooth muscle cell layers from 16-week-old mice and consisted of three independent experiments, each with bladders pooled from three mice, for a total of 9 WT male, 9 WT female, 9 *Entpd1*^−/−^ male, and 9 *Entpd1*^−/−^ female mice. For this purpose, mice were anesthetized by isoflurane inhalation and perfused with phosphate-buffered saline (PBS) following the method described by Lu et al. (2019) [41]. Briefly, approximately 24 mL of PBS was injected in the left ventricle of mouse heart while the right atrium was cut. After the lungs and liver were completely emptied of blood, the bladder was removed, and the smooth muscle layer was separated from the mucosa (epithelium and lamina propria) under a magnifying glass using pliers. RNA was isolated using the Trizol method according to the manufacturer’s recommendation (Invitrogen). Briefly, total RNA from male and female mice bladder smooth muscle layer was extracted with Trizol then quantified with a Biodrop spectrophotometer. The cDNA was synthesized with SuperScript III from 1 µg of total RNA with oligo (dT)_18_ as the primer, according to the instructions of the manufacturer (Invitrogen, Carlsbad, CA, USA). Primers specific for the ectonucleotidases (*Entpdl, Entpd2, Entpd3,* and *Entpd8*), ecto-5′-nucleotidase (*Nt5e*), P2X (*P2rx1-7*), P2Y (*P2ry1, P2ry2, P2ry4, P2ry6,* and *P2ry12-14*) and P1 receptors (*Adora 1, Adora2a, Adora2b,* and *Adora3*) were either designed by us and synthetized by Invitrogen (Carlsbad, CA, USA) or purchased from Qiagen (Toronto, ON, Canada), as detailed in Table 1. Prior to RT-qPCR of target genes, bladder smooth muscle layer purity was verified by quantifying *Krt7* and *Myh11*. For quantitative PCR, equal amounts of cDNA were run in duplicate and amplified in a final volume of 10 µL containing 9 µL of a mixture of 2X FastStart Universal SYBR Green Master Mix (Roche Diagnostics, Indianapolis, IN, USA), specific primers and RNase-DNase free water, and 1 µl of target cDNA. The mixture was incubated at 50 °C for 2 min, at 95 °C for 10 min, and then cycled 40 times at 95 °C for 15 s and at 60 °C for 1 min using the Applied Biosystems Prism 7900 Sequence Detector. For the negative controls, water was used as template. Standard curves were used to determine mRNA transcript copy number in individual reactions. Glyceraldehyde 3-phosphate dehydrogenase (*Gapdh*) or actin beta (*Actb*) was used as reference genes to normalize RNA quantities between samples.

### 2.7. Measurement of Bladder Strips Contraction

Mice were sacrificed by CO_2_ inhalation, and their bladders were excised and placed in ice-cold physiological salt solution containing Krebs buffer 118 mM NaCl, 4.7 mM KCl, 1.9 mM CaCl_2_, 1.2 mM KH_2_PO_4_, 1.2 mM MgSO_4_, 25 mM NaHCO_3_, and 11 mM dextrose (D-glucose). Two strips of approximately 4 mm long and 3 mm wide were prepared and mounted on a 5 mL vertical organ bath system connected to a Haake S3 heated circulating water bath (Thermo Scientific, Newington, NH, USA) and to an isometric transducer (Harvard Apparatus, Cambridge, MT, USA). Tissue contraction was recorded by a LKB Bromma 2210 recorder (Kipp and Zonen, Delft, Holland). The system was continuously bubbled with 95% O_2_ and 5% CO_2_. Bladders were set to a 1 g tension and left to equilibrate for 90 min. After equilibrating contraction with 10 µM carbachol, stimulation with nucleotides (ATP, ADP, UTP or UDP at 100 µM) or with 5-hydroxytryptamine (5HT) and the thromboxane analog U46619 as controls were performed. After recording each contraction for 5 min, the system was washed and was rebalanced for 30 min before the next stimulation. Where indicated apyrase at a concentration of 2 U/mL, or the same amount of boiled apyrase, were added to the strips 40 min before stimulation with the indicated nucleotides. For contraction experiments involving WT and *Entpd1*^−/−^ mice, 12-week-old mice (20 WT males, 20 *Entpd1*^−/−^ males, 15 WT females and 15 *Entpd1*^−/−^ females) were used. Fourteen-week-old mice (15 WT males, 15 *P2ry6*^−/−^ males, 10 WT females and 10 *P2ry6*^−/−^ females) were used for experiments with WT and *P2ry6*^−/−^ mice. For apyrase experiments, 12-week-old mice (4 WT males and 4 *Entpd1*^−/−^ males) were used.

### 2.8. Statistical Analysis

Results are expressed as mean ± standard error of mean (SEM). The statistical differences between mean values were assessed by two-way ANOVA followed by Tukey post-test for multiple comparisons. Statistical analysis was performed using GraphPad, Prism V7.0 (GraphPad Software San Diego, CA, USA). A value of *p* < 0.05 was considered statistically significant.

## 3. Results

### 3.1. Immunolocalization and Activity of Ectonucleotidases in Bladder SMCs

**Figure 1:**
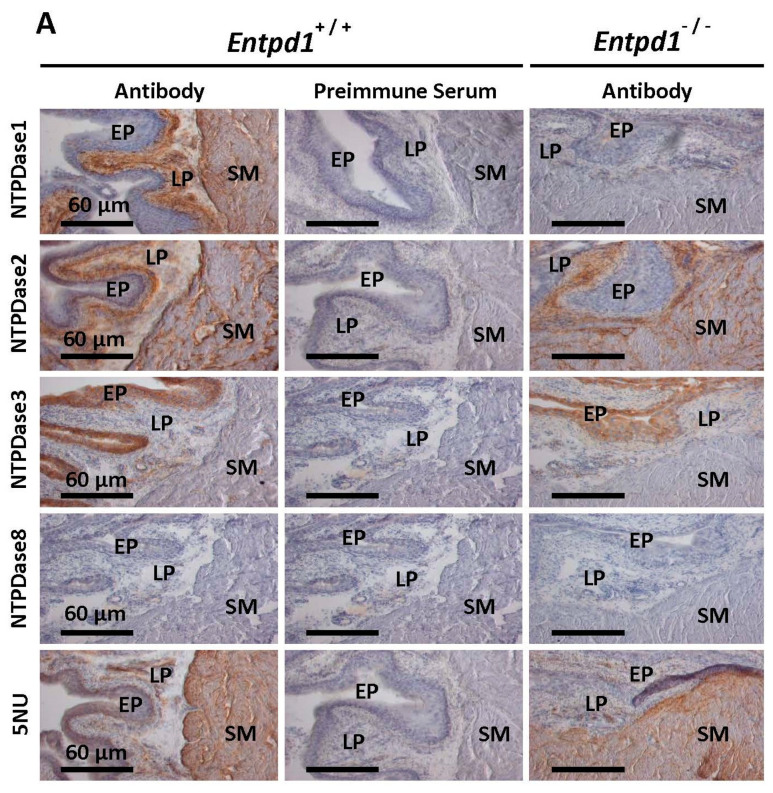

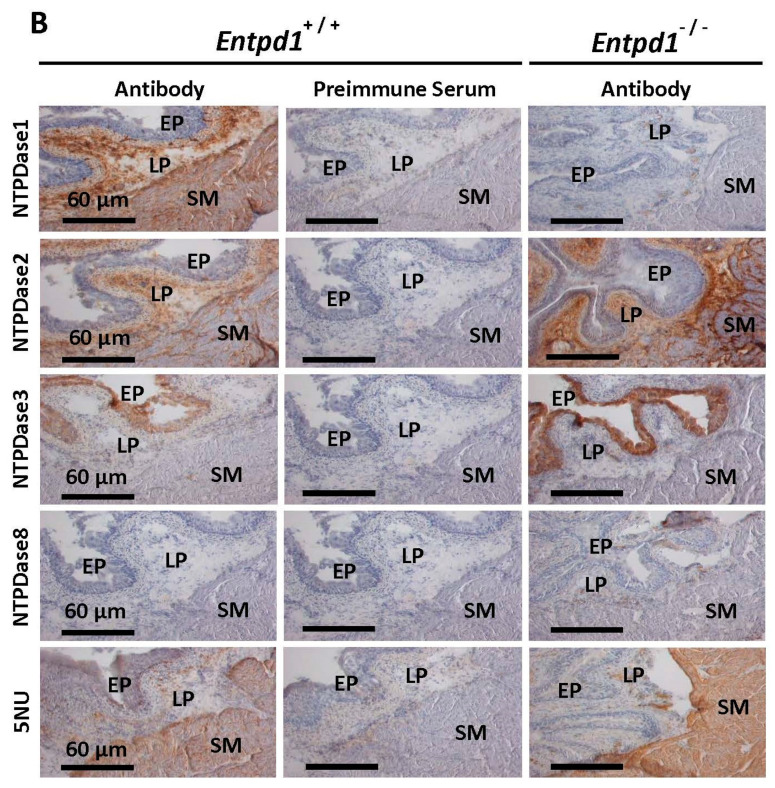
Immunolocalization of ectonucleotidases in male (**A**) and female (**B**) mouse bladder. SM: smooth muscle; LP: lamina propria. EP: epithelium. bar = 60 µm. *Entpd1*^+/+^: wild type mice. *Entpd1*^−/−^: NTPDase1 deficient mice. 5NU: ecto-5′-nucleotidase. Experiments were done on 3 mice of each group.

In mice bladder, NTPDase1 was localized at the surface of smooth muscle cells and in the lamina propria (Figure 1). No signal was observed in mice deficient in NTPDase1 (*Entpd1*^−/−^), confirming both the specificity of the antibody used against NTPDase1 and the absence of NTPDase1 in *Entpd1*^−/−^ mice. NTPDase2 was localized between smooth muscle fibers and in the lamina propria. There were no immunoreactions in the bladder SMCs when using antibodies to NTPDase3 and NTPDase8 (Figure 1). NTPDase3 immunolocalization was restricted to the epithelium while ecto-5′-nucleotidase was localized in the smooth muscle. A similar immunolocalization was observed for these 3 enzymes in male (A) and female (B) bladder.

**Figure 2:**
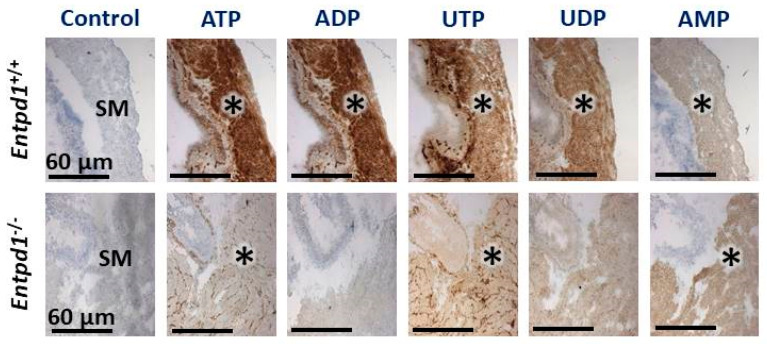
Comparative analysis of ectonucleotidase activity in the bladder of *Entpd1*^+/+^ (wild type) and *Entpd1*^−/−^ (nucleoside triphosphates diphosphohydrolase 1 (NTPDase1) deficient) female mice. SM (*): smooth muscle. bar = 60 µm. Control: no nucleotides. Adenosine 5′-triphosphate (ATP), adenosine 5′-diphosphate (ADP), uridine 5′-triphosphate (UTP), uridine 5′-diphosphate (UDP), and adenosine 5′-monophosphate (AMP) were used as substrate at a concentration of 100 µM. Experiments were done on three mice of each group.

**Figure 3:**
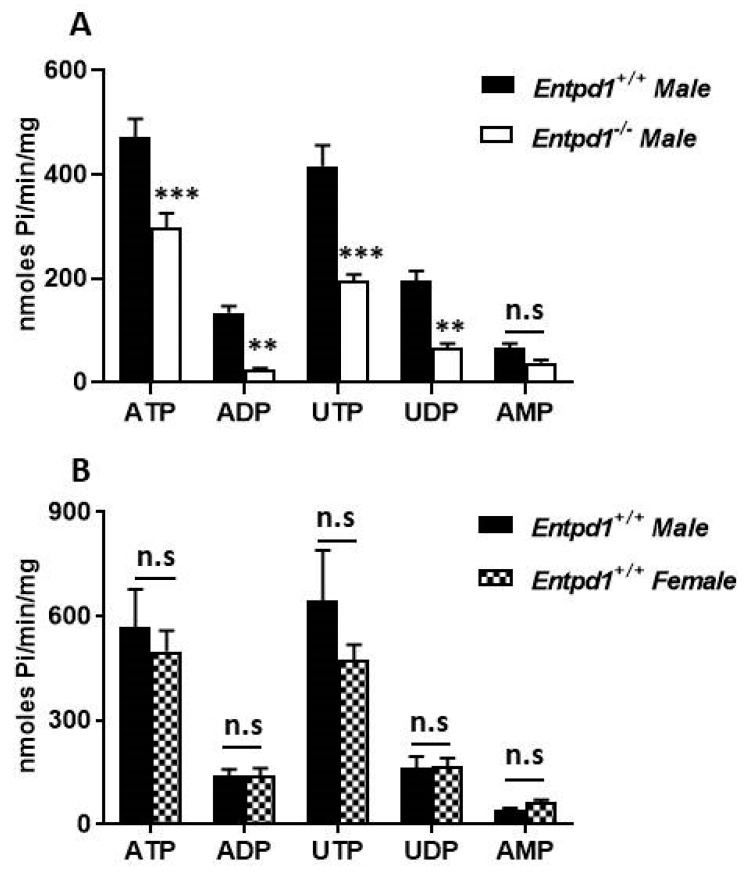
Hydrolysis of nucleotides in homogenates of male and female mouse bladders. Data presented are the mean ± SEM of seven experiments with homogenates from *Entpd1*^+/+^ (wild type) and *Entpd1*^−/−^ (NTPDase1 deficient) male bladders (**A**) and four experiments with homogenates from male and female *Entpd1*^+/+^ bladders (**B**), each in triplicate. Two symbols *p* < 0.01, three symbols *p* < 0.001. * Hydrolysis of *Entpd1*^−/−^ homogenates compared to that of wild type homogenates. n.s: not significant. ATP, ADP, UTP, UDP, and AMP were used as the substrate at a concentration of 500 µM.

Ectonucleotidase activity in WT female bladders revealed an intense hydrolysis of nucleotides by NTPDase1 which was detected as a brown precipitate in SMCs (asterisk) of the bladder for the four P2 receptor ligands: ATP, ADP, UTP, and UDP (Figure 2). ADPase and UDPase activities were absent in female *Entpd1*^−/−^ bladders. The remaining ATPase and UTPase activities between smooth muscle fibers (asterisk) in female *Entpd1*^−/−^ tissues, which is also present in WT, corresponds to NTPDase2 immunolabeling. Indeed, NTPDase2 has a low diphosphatase activity, especially when compared to NTPDase1 [29,42]. The activity of ecto-5′-nucleotidase which was revealed with its substrate AMP was also located at the surface of SMCs in both female WT and *Entpd1*^−/−^ tissues. This AMPase activity correlated with ecto-5′-nucleotidase immunolabelling. The nucleotidase activities were similar in male (data not shown) as in female bladders (Figure 2). The hydrolysis of the ectonucleotidase substrates ATP, ADP, UTP, and UDP was lower in the homogenates of male bladders deficient in NTPDase1 (Figure 3A), but not for the ecto-5′-nucleotidase substrate AMP, as expected. There were no significant differences in the hydrolysis of these 5 substrates in the homogenates from male and female WT bladders (Figure 3B).

### 3.2. Contractile Effect of Nucleotides is Increased in Entpd1^−/−^ Bladder Strips

**Figure 4:**
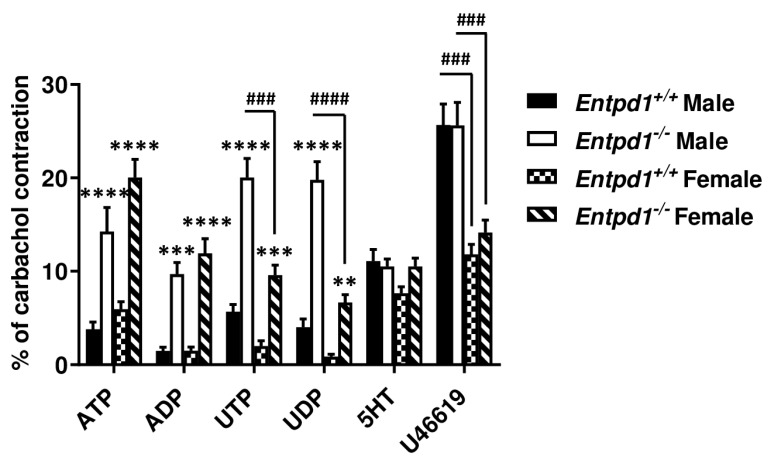
Contractile effect of extracellular nucleotides on male and female mouse bladder strips. Data presented are mean ± SEM of 20 contraction experiments with *Entpd1*^+/+^ (wild type) and *Entpd1*^−/−^ (NTPDase1 deficient) male bladders and 15 with *Entpd1*^+/+^ and *Entpd1*^−/−^ female bladders. Two symbols *p* < 0.01, three symbols *p* < 0.001, and four symbols *p* < 0.0001. * Contraction of *Entpd1*^−/−^ mice compared to that of wild type strips and # contraction of male strips compared to female strips of the same genotype. ATP, ADP, UTP, and UDP were used at a concentration of 100 µM, 5-hydroxytryptamine (5HT) at 10 µM, and U46619 at 100 nM.

We then questioned whether nucleotide could affect SMCs contraction in mouse bladder and if the dominant ectonucleotidase expressed in bladder SMCs, NTPDase1, could modulate their contractile effects. To address this question, we compared the contraction of WT bladders (*Entpd1*^+/+^) with that of tissues deficient for NTPDase1 expression (*Entpd1*^−/−^). As can be seen in Figure 4, The contraction caused by each of these four nucleotides was exacerbated in *Entpd1*^−/−^ bladders. Uracil nucleotides (UTP and UDP) produced the strongest contractions of male bladder strips. The latter contractions were significantly reduced in female bladders. The contractile effect of adenine nucleotides (ATP and ADP) was comparable in the bladders of both sexes. The non-nucleotide agonists 5-hydroxytryptamine (5HT) and the thromboxane A2 analogue U46619 activated contraction similarly in both genotypes showing that the smooth muscles in *Entpd1*^−/−^ bladders contracted normally, as in WT mice. There was a reduction in the contractile response to U46619 in strips from female bladders for both genotypes.

### 3.3. mRNA Expression of Ectonucleotidases and Nucleotide Receptors in Mouse Bladder Smooth Muscle Layer

**Figure 5:**
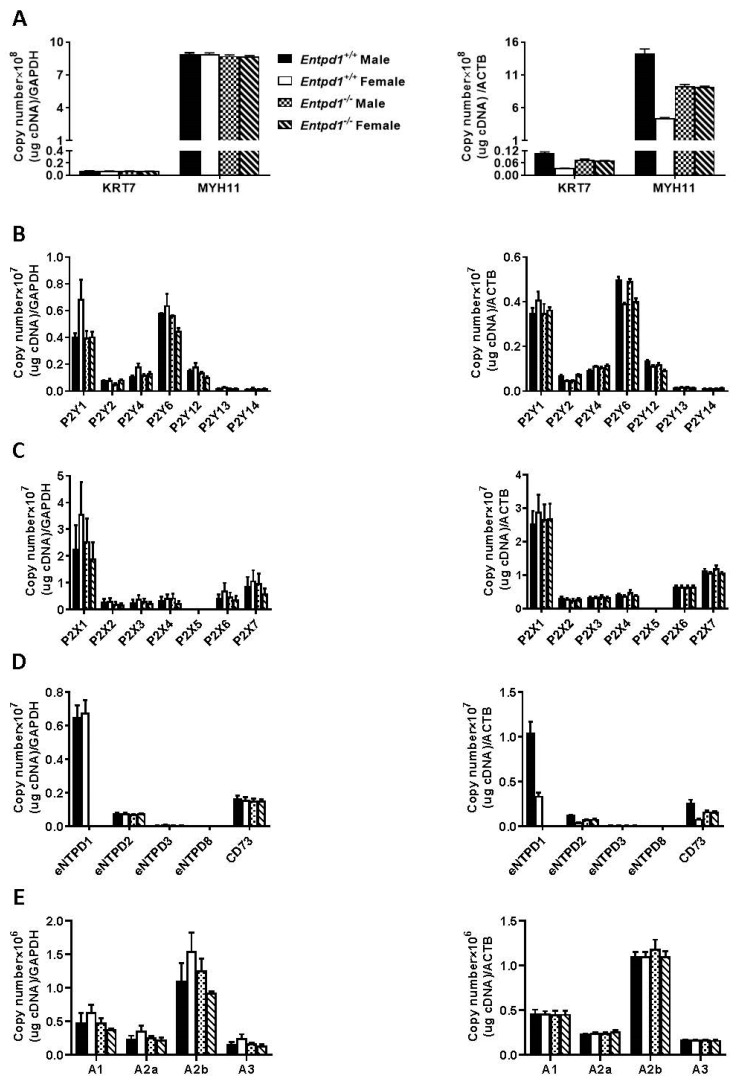
Gene expression profile in male and female bladder SMCs. SMCs were separated from the epithelial layer as mentioned in the “Materials and Methods” section. RNA was isolated from the SMC layer and the expression of the epithelial cell marker Krt7 and the SMC marker Myh11 (**A**), P2Y receptors (**B**), P2X receptors (**C**), ectonucleotidases (**D**), and P1 receptors (**E**) were quantified by RT-qPCR. Data are normalized to GAPDH (glyceraldehyde 3-phosphate dehydrogenase; **left** panels) and ACTB (actin beta; **right** panels) mRNA level. *Entpd1*^+/+^: wild type mice. *Entpd1*^−/−^: NTPDase1 deficient mice. Data presented are the means ± SEM of three independent experiments in duplicate each with SMCs pooled from three mice.

The data from Figure 4 suggest the implication of nucleotide receptors in these effects where the ectonucleotidase NTPDase1 plays a central role in the modulation of the process. We then measured the expression of these potential actors by RT-qPCR. First, we quantified *Krt7* (a marker of epithelial cells) and *Myh11* (a marker of SMCs) in our smooth muscle samples. As seen in Figure 5A, mRNA levels of *Krt7* were negligible when compared to *Myh11* for both sexes and both genotypes, demonstrating that the preparation of smooth muscles is exempt of epithelial cells. It is noteworthy that we perfused the animals before sacrifice to avoid blood cell contamination. We then quantified the expression of ectonucleotidases, as well as of the nucleotide receptors and of the adenosine receptors to find the potential receptor(s) involved in the contractile response to nucleotides. Among P2Y receptors, Figure 5B shows that P2Y_1_ and P2Y_6_ were the most highly expressed receptors at the mRNA levels, followed by P2Y_12_, P2Y_4_, and P2Y_2,_ respectively. A modest expression was measured for P2Y_13_ and P2Y_14_. Concerning P2X receptors, P2X1, was the most highly expressed receptor followed by P2X7, P2X6 and P2X4, P2X3, and P2X2, in decreasing order of mRNA expression levels. No P2X5 expression was detected (Figure 5C). NTPDase1 and ecto-5′-nucleotidase showed the highest mRNA levels among the nucleotide hydrolyzing enzymes expressed followed by NTPDase2. Very low NTPDase3 expression level was measured while no NTPDase8 expression could be detected (Figure 5D). It is noteworthy to point out that NTPDase1 not only hydrolyzes the agonists of P2 receptors but, in tandem with ecto-5′-nucleotidase, also generates the P1 receptor agonist. We therefore also evaluated the mRNA expression of the P1 receptors. Among these receptors, the adenosine A2_B_ receptor was the most highly expressed adenosine receptor, followed by A_1_, A_2A_, and A_3_, in decreased order of expression (Figure 5E). It is noteworthy to mention that *Gapdh* was the most stable control in bladder smooth muscle layer for the cell markers and for the ectonucleotidases expression (Figure 5A,D) while *ActB* was more stable for P2Y, P2X, and P1 genes expression (Figure 5B,C,E).

### 3.4. Implication of P2Y_6_ Receptor in the Contractile Response of Bladder SMCs

**Figure 6:**
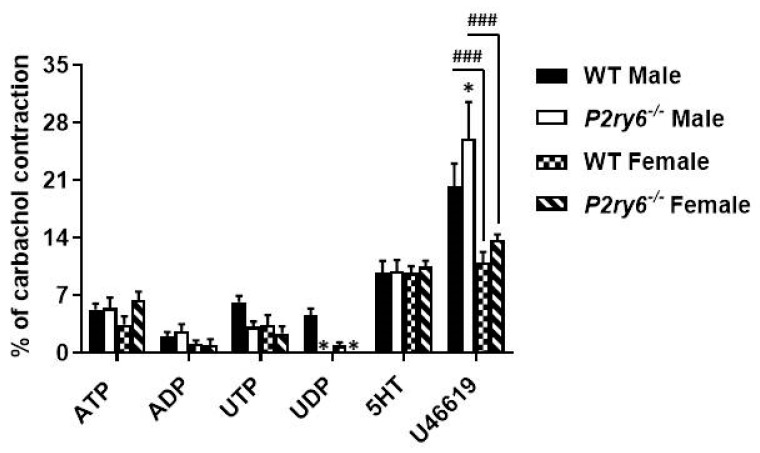
Contractile effect of extracellular nucleotides on male and female strips from *P2ry6*^−/−^ mouse bladders. Data are presented as the mean ± SEM of 15 contraction experiments with strips from WT (wild type) and *P2ry6*^−/−^ (P2Y_6_ receptor knock-out) male bladders and 10 with strips from WT and *P2ry6*^−/−^ female bladders. One symbol *p* < 0.05, 3 symbols *p* < 0.001. * Contraction of *P2ry6*^−/−^ bladders compared to WT; # Contraction of male strips compared to female strips of the same genotype. ATP, ADP, UTP, and UDP were used at the concentration of 100 µM, 5HT at 10 µM, and U46619 at 100 nM.

Figure 4 showed an increased contraction in *Entpd1*^−/−^ bladder SMCs for the four nucleotides tested. Among them, UTP and UDP induced the strongest contractions. As both nucleotides are agonists of P2Y_6_ receptor in mice [19], we investigated whether this receptor was involved in these effects. We therefore compared contraction of strips from WT and P2Y_6_ KO bladders. While the contractile responses to ATP and ADP were similar in P2Y_6_ deficient bladders of both sexes, when compared to WT bladders, the effect of the P2Y_6_ agonist UDP was totally abrogated, confirming that this receptor was implicated in the response observed in Figure 4. Although the response to UTP was also reduced in strips from *P2ry6*^−/−^ bladders, it was not significantly different from WT strips (Figure 6). This implies that P2Y_2_ and/or P2Y_4_ were also responsible for part of this contraction as both receptors are also activated by UTP. Therefore, these data suggest the implication of P2Y_6_ in the responses to UDP and that UTP activate contractions via P2Y_6_ as well as via other P2 receptors (P2Y_2_ and/or P2Y_4_). Strips from male bladders contracted significantly more in the presence of the thromboxane analogue (U46619) when compared to females in both genotypes (WT and *P2rY6*^−/−^).

### 3.5. Apyrase Reversed the Increased Contraction in Entpd1^−/−^ Bladders

**Figure 7:**
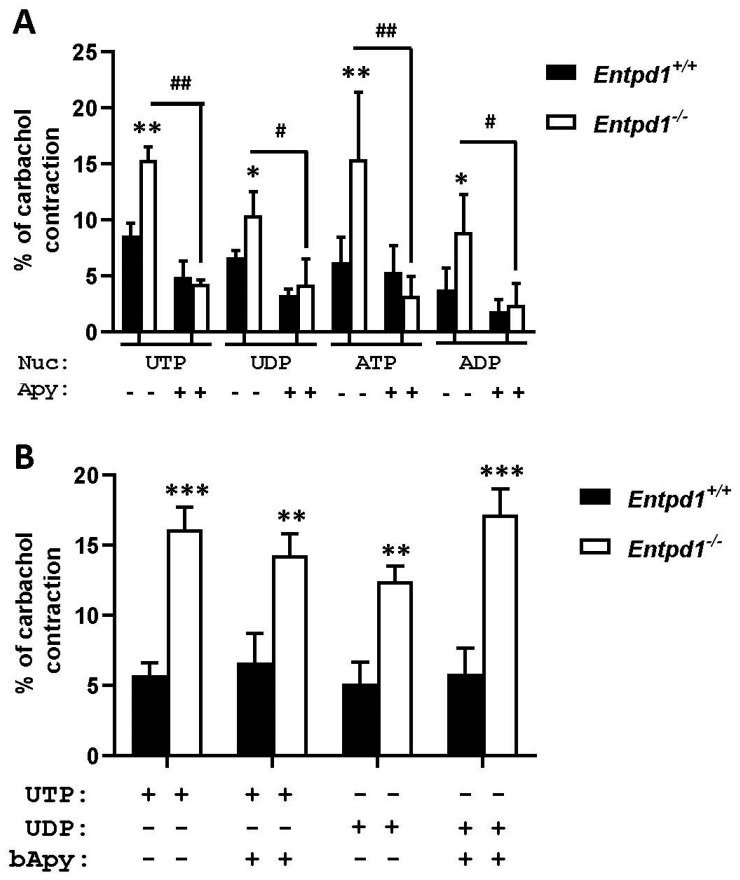
Effect of apyrase (Apy, 2 U/mL) (**A**) and the same amount of boiled apyrase (bApy) (**B**) on contraction induced by 100 µM ATP, ADP, UTP, or UDP on strips from male mouse bladders. Data presented are the mean ± SEM of four contraction experiments with strips from different mice. One symbol *p* < 0.05, two symbols *p* < 0.01, and three symbols *p* < 0.001. * Contraction in *Entpd1*^−/−^ bladders compared to contraction in WT; # Contraction in *Entpd1*^−/−^ bladders without apyrase compared to *Entpd1*^−/−^ bladders in presence of apyrase. There was no significant difference for WT bladders in presence or absence of apyrase or boiled apyrase.

To confirm the role of NTPDase1 nucleotidase activity in the contraction process, we used 2 U/mL of potato apyrase, a commercially available enzyme that hydrolyses nucleotides similarly as does NTPDase1. As can be seen in Figure 7A, responses to ATP, ADP, UTP, and UDP were significantly reduced in *Entpd1*^−/−^ bladders in presence of apyrase. Heat inactivated apyrase did not have any effects on nucleotide induced contraction in either *Entpd1*^+/+^ or *Entpd1*^−/−^ bladders (Figure 7B), confirming that its effect was due to nucleotidase activity.

## 4. Discussion

Over the past decades, NTPDase1 was reported to regulate several vascular functions such as thrombosis [30], angiogenesis [43], and vascular permeability [44]. In this study, we show that NTPDase1 was localized at the surface of bladder SMCs as demonstrated by immunohistochemistry (Figure 1). This localization correlated with high hydrolytic activity detected in wild type SMCs for ATP, ADP, UTP, and UDP which was absent at the surface of *Entpd1^−/−^* bladder (Figure 2). Accordingly, specific activity in tissue homogenates was reduced in *Entpd1^−/−^* bladder (Figure 3). Therefore, our results presented in this work demonstrate that NTPDase1 is the dominant ectonucleotidase in bladder SMCs in agreement with a previous study which reported NTPDase1 labeling by immunofluorescence in detrusor smooth muscle cells of mice bladder [45].

In absence of NTPDase1, we observed an enhanced contraction of bladder strips ex vivo (Figure 4). These nucleotide-dependent contractile effects appeared to be due to the activation of different receptors at the SMC surface. Based on our qPCR data, we found that P2Y_6_, P2Y_1_, and P2X1 were the major receptors expressed at the mRNA levels. P2Y_12_, P2Y_2_, and P2Y_4_ were also expressed at high levels (Figure 5). We also noted expression of, in decreasing order of expression, P2X7, P2X6, P2X4, P2X3, and P2X2 receptors which are potential candidates implicated in the contraction induced by ATP.

In the settings presented in this work, the strips from male bladders responded more strongly to UDP and UTP than to ADP and ATP, suggesting the implication of P2Y_6_ receptors (Figure 4). Experiments conducted with *P2ry6^−/−^* bladders confirmed the implication of this receptor in the contraction process (Figure 6) as the contractile effect of the P2Y_6_ agonist UDP was totally abrogated. This response was reduced in female bladder while ATP and ADP-induced contractions were comparable between bladders of both sexes. This suggests a differential sex-regulated contribution of P2Y_6_, versus the other P2 receptor types (e.g., P2Y_12_ and P2X1) also involved in SMC contraction in bladder, between male and females.

We found that apyrase reversed the response observed in *Entpd1^−/−^* bladder (Figure 7A). This result confirmed that nucleotides induced contraction via the activation of P2 receptors and it also confirmed the role of NTPDase1 biochemical activity in the modulation of contraction.

The implication of P2Y_6_ in the contraction process has also been reported in vascular smooth muscles. Kauffenstein et al. (2010) reported an enhanced UTP and UDP dependent contraction in *Entpd1*^−/−^ mice aorta and mesenteric resistance arteries via P2Y_6_ receptors [19,46]. Yu et al. (2013) demonstrated that the absence of NTPDase1 led to an increased P2Y_6_ contractility of mice bladder smooth muscle upon activation with either UDP or MRS 2693, two specific agonists of this receptor [47]. This was confirmed by the fact that when cholinergic component was blocked with atropine, MRS 2693 significantly increased electric field stimulation induced purinergic contraction force up to 45%. This potentiation was blunted by MRS 2578, a specific P2Y_6_ receptor antagonist. Carneiro et al. (2014) reported that the activation of P2Y_6_ receptors following instillation of UDP or PSB0474, another P2Y_6_ receptor agonist, into the bladder of anesthetized rats, increased the voiding frequency by releasing ATP from the urothelium [48]. More recently, Kira et al. showed that P2Y_6_ receptor deficiency increased micturition frequency and attenuated sustained mice bladder contractility [49]. Hence, P2Y_6_ enhance bladder contraction through direct and indirect (P2X signaling) mechanisms. Difference in the activation of P2Y_6_ receptor due to sex was not investigated in these studies.

P2Y_2_ and/or P2Y_4_ were also implicated as UTP response was not completely abrogated in *P2ry6^−/−^* bladders. The response obtained with ADP in this study agrees with what was observed by Yu et al. (2014) who reported that ADP induced contraction in mice via P2Y_12_ receptors [50]. As high expression of the ADP receptor P2Y_1_ was also found by qPCR (Figure 5B), the role of this receptor in SMC contraction could also be possible. However, Yu et al. (2014) reported that MRS 2365, a selective P2Y_1_ receptor agonist, was ineffective at eliciting bladder SMCs contraction [50]. This was corroborated with MRS 2500, a selective P2Y_1_ receptor antagonist, which did not inhibit ADP-induced bladder SMCs contraction. They concluded that P2Y_1_ receptor was not implicated in bladder contraction.

Bladder contractility elicited by ATP has been described for nearly 50 years [51,52]. ATP-induced contraction reported in this study is in line with other studies which have reported that this contractile effect was due to P2X1 receptor activation [53,54]. We also found that other P2X receptors were expressed in the bladder (Figure 5C) and our results do not exclude that these receptors could also participate in the contraction. In agreement, Kennedy et al. suggested the implication of P2X1/P2X4 heteromultimer in the contraction of mice and guinea pig bladders [55]. This result was, however, in disagreement with the conclusion of Yu et al. who localized P2X4 receptor by immunofluorescence between muscle bundles and associated with the vasculature [56]. They also showed that P2X4 null mice maintained macroscopic and voiding function similarities with wild type mice [56]. Altogether, these data suggest that P2X1 receptor is the main P2X subtype implicated in ATP-induced bladder contractility and they suggest that other P2X subtypes might play a role in other bladder functions.

The increase in the contractile response to nucleotides in *Entpd1^−/−^* bladder reveals a crucial role for NTPDase1 nucleotidase activity to prevent nucleotide receptors activation. This increased nucleotide contractility in *Entpd1^−/−^* bladder is in line with the previous report of Kauffenstein et al. (2010) in the vasculature [19]. Other studies with *Entpd1^−/−^* mice have however revealed P2 receptors desensitization, suggesting that NTPDase1 can also prevent desensitization and can therefore allow some P2 receptor activation in the appropriate time [30,43,57,58]. Thus, depending on the type of receptors involved, NTPDase1 may play different roles: for easily desensitized receptors such as P2Y_1_ and P2X1, NTPDase1 can prevent receptor desensitization. In contrast, for poorly desensitized receptors such as P2Y_6_, as evidenced here and by Kauffenstein et al. (2010), NTPDase1 limits their activation and its inhibition/deletion reveals full contractile effects of the latter receptor. NTPDase1 appears therefore as essential in terminating P2Y_6_ receptor signaling, especially considering the few uracil nucleotides metabolizing enzymes apart from NTPDases [25].

Taken together, our data suggest that NTPDase1 modulates mouse bladder SMCs contraction by regulating the activation of several nucleotide receptors.

A peculiar finding made during this study was the decrease in UTP and UDP responses in female bladder compared to male bladder. This indicates sex difference in mice bladder smooth muscle contraction in response to stimulating nucleotides. We suspected a reduced expression of P2Y_6_ in female bladders. This was however not confirmed in our RT-qPCR results for P2Y receptors (Figure 5B). It is likely that despite similar mRNA levels, protein expression of P2Y_6_ or coupling to intracellular signaling pathways may differ from male to female. Notably, P2Y_6_ and thromboxane A2 receptors share Rho signaling pathway components [46,59,60]. Sex differences in this pathway may explain both UTP/UDP and U46619 (thromboxane analogue) enhanced contraction in male bladder.

Such difference in response to stimulating molecules between sexes has been extensively demonstrated in vascular functions of the aorta, coronary, mesenteric, and renal arteries from various species including mice, rat, and pigs [61,62,63]. The reduced response to UTP and UDP (Figure 4) correlates with the results of Ma et al. who found a reduction in the contraction of SMCs of female rat aorta stimulated by phenylephrine, angiotensin, and depolarizing KCl in comparison to male aorta [64]. They associated this reduction to an increased estrogen receptor expression in the female tissue.

More recently, Caiazzo et al. found a reduced in vitro response to ADP of female rat’s platelets compared to male platelets and concluded that this was due to an increased expression and activity of NTPDase1 [65]. Conversely, ecto-5′-nucleotidase expression did not follow the same pattern as comparable expression levels were found between male and female platelets [65]. This last enzyme was reported to control extracellular adenosine level in a murine model over-expressing NTPDase1, thereby, reducing ex vivo platelet aggregation and exerting antithrombotic, anti-inflammatory, and cardio protective effects through adenosine receptors signaling [66,67].

Regarding bladder function, sex differences in the pharmacology and physiology of lower urinary tract has been investigated at the neurogenic level and has been mainly attributed to differential expression of muscarinic and adrenergic receptors between male and female in humans, mice, rats, and rabbits [68]. Kamei et al. investigated how sex and age affects bladder function and gene expression in mice. They reported storage and voiding dysfunctions with ageing in both sexes. They also pointed out weaker detrusor strips contractile response from aged mice, decreased mRNA expression of bladder tissue for M3 muscarinic receptors in aged males and β2-adrenoreceptors in aged females. They concluded that these age-related impairments were generally more severe in males [69].

Altogether, these data show that bladder contractility obeys different rules in males and in females. In addition, our data suggest that NTPDase1 and P2Y_6_ receptor signaling contribute to these differences.

In this work, we focused our attention on nucleotide signaling in the activation of contraction. The role of adenosine, which has been shown to inhibit human and mice bladder SMCs contractility [70,71] was not investigated in this work. Nevertheless, our work suggests the potential contribution of ecto-5′-nucleotidase in these effects. Indeed, we have demonstrated the presence of ecto-5′-nucleotidase, alongside NTPDase1, by immunohistochemistry, by enzymatic histochemistry, and by RT-qPCR (Figure 5D). We have also shown the expression of adenosine receptors by RT-qPCR (Figure 5E). It is worth remembering that NTPDase1 not only terminates nucleoside triphosphate signaling but also generates AMP, which is subsequently converted to adenosine by ecto-5′-nucleotidase, which can finally activate P1 receptors and modulate bladder SMCs contraction or dilation.

## 5. Conclusions

In this work, we have shown that NTPDase1 is the major ectonucleotidase hydrolyzing nucleotides at the surface of male and female mice bladder SMCs where it reduced nucleotide-induced contraction by regulating their concentration levels. In absence of NTPDase1 in *Entpd1^−/−^* bladder, an increased contraction was observed in response to stimulating nucleotides. The nucleotide receptor P2Y_6_ was the most important nucleotide receptor in these effects in male. Interestingly, the P2Y_6_-dependent constrictor response was significantly reduced in female *Entpd1^−/−^* bladder when compared to male. P2Y_6_, P2Y_1_, and P2X1 were the most abundantly expressed P2 receptors at the mRNA level, followed by several other P2 receptors, among which P2Y_12_, P2Y_2_, P2Y_4_, which also appeared to contribute to SMCs contraction. The increased contractions observed in *Entpd1^−/−^* bladders were reversed by potato apyrase, confirming the implication of NTPDase1 biochemical activity in the regulation of nucleotide-dependent constrictor effects. The results presented in this study suggest that changes in NTPDase1 enzymatic activity due to pathological conditions like lower urinary tract symptoms could contribute to bladder contraction disorders.

## Figures and Tables

**Table 1:** qRT-PCR primers.

Gene	Forward Primer	Reverse Primer	Amplicon (bp)
*Krt7*	Qiagen	Qiagen	134
*Myh11*	Qiagen	Qiagen	112
*Gapdh*	CCA TCA CCA TCT TCC AGG AG	GTG GTT CAC ACC CAT CAC AA	194
*Actb*	GGC TGT ATT CCC CTC CAT CG	CCA GTT GGT AAC AAT GCC ATG T	154
*Entpdl*	AGC TGC CCC TTA TGG AAG AT	TCA GTC CCA CAG CAA TCA AA	123
*Entpd2*	TTC CTG GGA TGT CAG GTC TC	GTC TCT GGT GCT TGC CTT TC	132
*Entpd3*	ACC TGT CCC GTG CTT AAA TG	AGA CAG AGT GAA GCC CCT GA	183
*Entpd8*	Qiagen	Qiagen	146
*Nt5e/CD73*	CAG GAA ATC CAC CTT CCA AA	AAC CTT CAG GTA GCC CAG GT	128
*P2ry1*	TCG TGT CTC CAT TCT GCT TG	CGA CAG GGT TTA TGC CAC TT	218
*P2ry2*	TGA CGA CTC AAG ACG GAC AG	GTC CCC TAC AGC TCC CCT AC	108
*P2ry4*	AGA CGG GCC TGA TGT GTA TC	AGG TTC ACA TGC CCT GTA CC	126
*P2ry6*	GGT AGC GCT GGA AGC TAA TG	TTT CAA GCG ACT GCT GCT AA	308
*P2ry12*	GGC AGC CTT GAG TGT TCT TC	ATA ACG TGC TAC CCG ACC TG	130
*P2ry13*	ATA GAG AAC CGG GAA CAG CA	CAA AAC AAA GCT GAT GCT CG	115
*P2ry14*	TTT TGT CGT CTG CTT TGT GC	GCA GCC GAG AGT AGC AGA GT	135
*P2rx1*	CAA CTG TGT GCC CTT CAA TG	GGT ACC ATT CAC CTC CTC CA	114
*Pr2x2*	GCT GGG CTT CAT TGT AGA GC	CCT GTC CAT GCA CAA TAA CG	281
*P2rx3*	ATT TCC TCA AAG GGG CTG AT	GTT CTG CAG CCC AAG GAT AA	204
*P2rx4*	CAC AAC GTG TCT CCT GGC TA	GCC TTT CCA AAC ACG ATG AT	125
*P2rx6*	TCA CCC GCT AAC CCT GTT AC	TAG TCC CGC TGA AGC TTT GT	242
*P2rx7*	AAT CGG TGT GTT TCC TTT GG	CCG GGT GAC TTT GTT TGT CT	165
*Adora1*	GTG ATT TGG GCT GTG AAG GT	AGT AGG TCT GTG GCC CAA TG	142
*Adora2a*	TCA ACA GCA ACC TGC AGA AC	GGC TGA AGA TGG AAC TCT GC	186
*Adora2b*	TCT GGC CTT TTG GAG AAG AA	TTT CCG GAA TCA ATT CAA GC	246
*Adora3*	TGT GGA GGG AGT CTC GTC TT	TCC TTC TGT TCC CCA CAT TC	101

## Data Availability

All data supporting reported results in this study are included in the manuscript or uploaded as Figures Zip file during the submission.

## Abbreviations

SMC	Smooth muscle cell
ATP	Adenosine 5′-triphosphate
ADP	Adenosine 5′-diphosphate
UTP	Uridine 5′-triphosphate
UDP	Uridine 5′-diphosphate
AMP	Adenosine 5′-monophosphate
UMP	Uridine 5′-monophosphate
RT-qPCR	Quantitative Real time polymerase chain reaction
E-NTPDase	Ecto-nucleoside triphosphate diphosphohydrolase
OCT	Optimal cutting temperature
Pi	Inorganic phosphate
SEM	Standard error of mean
5HT	5-hydroxytryptamine
U46619	Thromboxane A2 analogue
GAPDH	Glyceraldehyde 3-phosphate dehydrogenase
ACTB	Actin beta
